# Amylopectin biosynthetic enzymes from developing rice seed form enzymatically active protein complexes

**DOI:** 10.1093/jxb/erv212

**Published:** 2015-05-15

**Authors:** Naoko Crofts, Natsuko Abe, Naoko F. Oitome, Ryo Matsushima, Mari Hayashi, Ian J. Tetlow, Michael J. Emes, Yasunori Nakamura, Naoko Fujita

**Affiliations:** ^1^Department of Biological Production, Akita Prefectural University, 241–438 Kaidobata-Nishi, Shimoshinjo-Nakano, Akita city, Akita 010-0195, Japan; ^2^Institute of Plant Sciences and Resources, Okayama University, Chuo 2–20–1, Kurashiki city, Okayama 710-0046, Japan; ^3^Department of Molecular and Cellular Biology, College of Biological Science, University of Guelph, Guelph, Ontario, N1G 2W1, Canada

**Keywords:** Amylopectin, endosperm, glucan, protein–protein interaction, rice, starch, starch synthesis

## Abstract

Amylopectin is a highly branched, organized cluster of glucose polymers, and the major component of rice starch. Synthesis of amylopectin requires fine co-ordination between elongation of glucose polymers by soluble starch synthases (SSs), generation of branches by branching enzymes (BEs), and removal of misplaced branches by debranching enzymes (DBEs). Among the various isozymes having a role in amylopectin biosynthesis, limited numbers of SS and BE isozymes have been demonstrated to interact via protein–protein interactions in maize and wheat amyloplasts. This study investigated whether protein–protein interactions are also found in rice endosperm, as well as exploring differences between species. Gel permeation chromatography of developing rice endosperm extracts revealed that all 10 starch biosynthetic enzymes analysed were present at larger molecular weights than their respective monomeric sizes. SSIIa, SSIIIa, SSIVb, BEI, BEIIb, and PUL co-eluted at mass sizes >700kDa, and SSI, SSIIa, BEIIb, ISA1, PUL, and Pho1 co-eluted at 200–400kDa. Zymogram analyses showed that SSI, SSIIIa, BEI, BEIIa, BEIIb, ISA1, PUL, and Pho1 eluted in high molecular weight fractions were active. Comprehensive co-immunoprecipitation analyses revealed associations of SSs–BEs, and, among BE isozymes, BEIIa–Pho1, and pullulanase-type DBE–BEI interactions. Blue-native-PAGE zymogram analyses confirmed the glucan-synthesizing activity of protein complexes. These results suggest that some rice starch biosynthetic isozymes are physically associated with each other and form active protein complexes. Detailed analyses of these complexes will shed light on the mechanisms controlling the unique branch and cluster structure of amylopectin, and the physicochemical properties of starch.

## Highlight

Starch biosynthetic enzymes in rice endosperm are physically associated with each other and form enzymatically active multiple protein–protein complexes, several of which were common to cereals while others were unique.

## Introduction

Rice is one of the most important crops grown in Asian and African countries as it feeds more than half of the world population (Fairhurst and Dobermann, 2002; Awika, 2011). The applications of rice starch are varied, and usage has been determined by the unique physicochemical properties found in different cultivars of rice which are predominantly affected by starch structure (Burrell, 2003; Singh *et al.*, 2006).

Starch is composed of glucose polymers of linear amylose and frequently branched semi-crystalline amylopectin. Amylopectin is the major component of rice starch, accounting for >80% (Juliano *et al.*, 1981; Vandepute and Delcour, 2004; Pandey *et al.*, 2012). The chain-length and positioning of glucan branches in amylopectin directly influence the physicochemical properties such as gelatinization and pasting temperatures, stickiness, and ease of retrogradation (Jane *et al.*, 1999; Han and Hamaker, 2001; Lii *et al.*, 2004). Therefore, understanding the mechanisms which control amylopectin branch structure is important for a number of applications.

Amylopectin is synthesized by the orchestrated roles of at least four distinct classes of starch biosynthetic enzymes, each of which is composed of multiple isozymes (Nakamura, 2002; Tian *et al.*, 2009; Jeon *et al.*, 2010; Fujita, 2014). Starch synthases (SSs; EC 2.4.1.21) elongate α-1,4-linked linear glucan polymers using ADP-glucose as a substrate. Starch branching enzymes (BEs; EC 2.4.1.18) produce α-1,6-linked branches and are crucial in amylopectin formation as they are the sole enzymes forming branches in amylopectin. Starch debranching enzymes (DBEs) such as isoamylase (ISA; EC 3.2.1.68) and pullulanase (PUL; EC 3.2.1.41) hydrolyse and remove α-1,6-linked branches to give water-insoluble properties of organized amylopectin structure (reviewed by Nakamura, 2002; Jeon *et al.*, 2010; Hennen-Bierwagen *et al.*, 2012). In addition to these three classes of enzymes, starch phosphorylase (Pho; EC 2.4.1.1) is thought to be involved in the initiation steps of starch biosynthesis, elongating α-1,4-linked glucan polymers using glucose 1-phosphate (G1P) as a substrate (Satoh *et al.*, 2008; Jeon *et al.*, 2010). Temporal and spatial co-ordination of these four classes of enzymes (SSs, BEs, DBEs, and Pho) during amylopectin synthesis must be critical in order to convert large amounts of photosynthetic products to form the organized cluster structure of insoluble amylopectin, and to store them as starch granules in amyloplasts of rice endosperm. Understanding the interaction among the starch biosynthetic enzymes in rice may provide new targets for improving the quality and yield of rice grains.

The rice genome encodes 11 isozymes of SSs, three isozymes of BEs, four isozymes of DBEs, and two isozymes of Pho (Ohdan *et al.*, 2005). Each isozyme exhibits some glucan substrate specificity, but functional redundancy has also been observed through the analyses of mutants lacking specific starch biosynthetic enzymes(s) (reviewed by Fujita, 2014). SSI elongates short chains of amylopectin generated by BEIIb (Fujita *et al.*, 2006, 2008; Abe *et al.*, 2014; Nakamura *et al.*, 2014). The product is further extended by SSIIa and/or SSIIIa, although SSIIa is catalytically inactive in typical japonica rice (Umemoto *et al.*, 2002; Nakamura *et al.*, 2005; Bao *et al.*, 2006; Yu *et al.*, 2011). SSIIIa generates long chains connecting multiple clusters of amylopectin (Fujita et al., 2007, 2011; Ryoo *et al.*, 2007; Hanashiro *et al.*, 2011). Granule bound starch synthase I (GBSSI) is primarily involved in amylose biosynthesis (Wang *et al.*, 1995; Cai *et al.*, 1998; Terada *et al.*, 2000; Itoh *et al.*, 2003), but it is also involved in elongation of extra long chains of amylopectin (Takeda *et al.*, 1987; Hanashiro *et al.*, 2008). BE isozymes (BEI, BEIIa, and BEIIb) are highly expressed in rice, but only BEIIb is endosperm specific (Mizuno *et al.*, 1993; Ohdan *et al.*, 2005). BEIIb-deficient rice lines show an opaque seed phenotype, a substantial decrease in the number of short glucan chains, and an increase in long chains of amylopectin, resulting in altered physicochemical properties and a change in crystallinity from A type to B type (Nishi *et al.*, 2001; Tanaka *et al.*, 2004; Butardo *et al.*, 2011; Abe *et al*., 2013). In contrast, loss of either BEI or BEIIa does not alter seed morphology, although loss of BEI affects amylopectin branch structure, and BEI is suggested to form longer branches within amylopectin (Satoh *et al.*, 2003; Nakamura *et al.*, 2002, 2010).

DBE isozymes including ISA1, ISA2, and PUL, but not ISA3, are expressed in developing rice endosperm (Ohdan *et al.*, 2005), ISA1 being particularly important for amylopectin formation (kubo *et al.*, 1999; Utsumi *et al.*, 2011). ISA1-deficient mutants have sugary seed phenotypes which accumulate randomly branched water-soluble phytoglycogen, instead of amylopectin (Wong *et al.*, 2003; Kubo *et al.*, 2005).

There are two isoforms of Pho in the rice genome. Pho1 (or PhoL) is the plant-specific isozyme which is involved in starch synthesis, and is localized to the plastid of the developing endosperm in rice (Satoh *et al.*, 2008), whereas Pho2 is localized in the cytosol and is probably involved in α-glucan metabolism (Hwang *et al.*, 2010). Functional and mutually synergistic relationships between BEs and Pho1, and BEs and SSI, have been observed *in vitro* using recombinant rice starch biosynthetic enzymes (Nakamura *et al.*, 2012, 2014).

Protein–protein interactions among certain SS and BE isozymes have been shown using amyloplasts isolated from developing seeds of wheat (Tetlow *et al.*, 2004, 2008), maize (Hennen-Bierwagen *et al.*, 2008; Liu *et al.*, 2009*a*, 2012*a*, *b*), and barley (Ahmed *et al.*, 2015). The phosphorylation-dependent, trimeric complex formed between SSI, SSIIa, and BEIIb in maize is one of the best studied and characterized protein complexes among starch biosynthetic enzymes to date (Liu *et al.*, 2009*a*, 2012*a*, *b*; Makhmoudova *et al.*, 2014). Interactions between BEI and BEIIb, and association of SSIII with several other proteins including pyruvate orthophosphate dikinase (PPDK), BEIIb, and AGPase, have also been demonstrated (Hennen-Bierwagen *et al.*, 2009; Liu *et al.*, 2009*a*). In addition, interaction of ISA with a carbohydrate-binding module (CBM)-containing FLO6 protein was recently shown (Peng *et al.*, 2014).

Given current understanding, this study investigated whether protein–protein interactions are also found in rice endosperm, as well as investigating differences between species. The aims of this study were therefore to investigate the formation of starch biosynthetic isozyme complexes using wild-type japonica rice, which possesses inactive SSIIa and lower GBSSI expression levels compared with indica rice, and to determine the glucan-synthesizing ability of enzyme complexes of specific molecular weight.

## Materials and methods

### Plant material

*Oryza sativa* L. *japonica*, cv. Nipponbare plants were grown in the experimental field of Akita Prefectural University during the summer months under natural light conditions. Developing seeds from 10–14 days after flowering (DAF) were stored at –30 °C. Husks and seed coats were removed before use.

### Preparation of total, soluble, and insoluble protein extracts from developing rice endosperm

Eight endosperms, weighing ~13mg per grain and a total of 100mg, were used for each extraction. Total protein was extracted with 9 vols (w/v) of denaturing buffer containing 0.125M TRIS-HCl, pH 6.8, 8M urea, 4% SDS, and 5% β-mercaptoethanol. Samples were extracted overnight at room temperature, centrifuged at 20 000 *g* to remove gelatinized starch and other particulate matter, and supernatants were used for SDS–PAGE and western blotting. Soluble proteins were extracted on ice with 9 vols (w/v) (three repeats with 3 vols) of extraction buffer, containing 10mM HEPES-KOH, pH 7.5, 100mM NaCl. After extraction, samples were centrifuged at 20 000 *g* at 4 °C for 10min. The residual pellet was extracted with 9 vols (w/v) of denaturing buffer as mentioned above and, following centrifugation, the supernatant was used to represent insoluble, starch granule-associated proteins.

### Generation of SSIIa and ISA1 peptide-specific antibodies, and SSIVb and BEIIa anti-bodies

Chemically synthesized, high-performance liquid chromatography (HPLC)-purified peptides conjugated with a keyhole limpet haemocyanin (KLH) tag were prepared by Funakoshi Co. Ltd. Amino acid sequence used for antigens were as follows. LLSGRDDDTPASRN corresponding to residues 154–168 of *Os*SSIIa (GenBank accession no. AF419099) and EPLVDTGKPAPYD corresponding to residues 750–762 of *Os*ISA1 (GenBank accession no. AB093426). Each peptide was injected weekly into a rabbit until the titre has reached the optimum for experiments. The full-length cDNA of OsSSIVb (GenBank accession no. AK067577) was amplified by PCR with the primers 5′-CAGCCTCCGCATCCGATTCC-3′ and 5′-TGTGGCATCAGCGGCCGCGTCAGAGAAAG-3′. The PCR product was cloned into NcoI and NotI sites of pET30c to add an N-terminal histidine tag. Plasmid was then digested with SalI and XhoI and self-ligated remove the catalytic domain. The partial cDNA of OsBEIIa (GenBank accession no. AB023498) was amplified with primers 5′TATTATGAATTCGGTGCTCCTGGGAAGGTGCTG 3′ and 5′TATTATCTCGAGCTCCACAGTTGGTTCATCAGC 3′. The PCR product was cloned into EcoRI and XhoI sites of pET30a.These plasmids were expressed in E. coli BL21 (DE3) containing the pKJE7 chaperone plasmid. Expressed proteins were separately purified by Ni-NTA resin (Qiagen) and run on SDS-PAGE. The Coomassie brilliant blue (CBB) stained proteins was excised and electro-eluted (Bio-Rad). The eluted proteins were injected to a rabbit for antibody generation.

### Gel permeation chromatography

A 700mg aliquot of endosperm was extracted in 1ml of gel filtration buffer containing 10mM HEPES-KOH, pH 7.5, 100mM NaCl, 10 μl ml^–1^ plant protease inhibitor cocktail (Sigma) and centrifuged at 20 000 *g* for 10min. The supernatant was filtered through 0.45 μm cellulose acetate to remove large particles and injected into a 500 μl sample loop, prior to fractionation by gel permeation chromatography (GPC) using Superdex 200 resin packed in a 10/300 column connected to an AKTAprime plus chromatography system (GE Healthcare) at 4 °C. The column was equilibrated with 10mM HEPES-KOH, pH 7.5, 100mM NaCl, and fractions eluted at 1ml min^–1^. Fractions of 2ml were collected and concentrated 25-fold using an Amicon Ultra 50K centrifugal filter unit (Merck Millipore) following the manufacturer’s instructions. Concentrated samples were mixed with one-third volume of native-PAGE sample buffer (0.625M TRIS-HCl, pH 7.0, 50% glycerol, 0.2% bromophenol blue). A 7.5 μl aliquot was applied per lane to the native (non-denaturing) PAGE (see next section). The residual samples were further supplemented with one-third volume of SDS–PAGE sample buffer (0.1M TRIS-HCl, pH 6.8, 10% SDS, 12% β-mercaptoethanol, 20% glycerol, 0.2% bromophenol blue), boiled, and 5 μl per lane subjected to 7.5% acrylamide SDS–PAGE (height 6cm, width 8.5cm, and thickness 1mm) at 25 mA, and western blotting.

### Native gel activity staining

SS-native-PAGE/activity staining was performed as described in Nishi *et al.* (2001) and Fujita *et al.* (2006). DBE native-PAGE/activity staining was performed as described in Fujita *et al.* (1999), and BE native-PAGE/activity staining was performed as described in Yamanouchi and Nakamura (1992).

### Immunoprecipitation

A 3g aliquot of endosperm was extracted with 9ml of 10mM HEPES-KOH, pH 7.5, 100mM NaCl, 1mM dithiothreitol (DTT), and 10 μl ml^–1^ plant protease inhibitor cocktail (Sigma). The extract was sieved through Miracloth. The residual materials were extracted again with 3ml of buffer (above) and sieved through the Miracloth. The pooled filtrates were centrifuged at 20 000 *g*, and 800 μl of each supernatant was mixed with 100 μl of isozyme-specific antibodies, or pre-immune serum as a control, for 1.5h at 4 °C. A 1000 μl aliquot of reconstituted 50% protein A–Sepharose resin (Sigma) was added and incubated for 1h at 4 °C. The resin was washed eight times with 10mM HEPES-KOH, pH 7.5, 100mM NaCl, 1mM DTT. Bound proteins were released by boiling for 10min in 150 μl of 1× SDS sample buffer containing 33mM TRIS-HCl, pH 6.8, 3.3% SDS, 4% β-mercaptoethanol, 6.6% (v/v) glycerol, 50mM DTT. After centrifugation at 12 000rpm for 2min, 10 μl of each supernatant was analysed by western blotting.

### Blue native (BN) PAGE

Endosperms were extracted with 3 vols (w/v) of 50mM BIS-TRIS, 6 N HCl, 50mM NaCl, 10% glycerol, 0.001% Ponceau S, and centrifuged at 20 000 *g* for 10min. The supernatant was supplemented with 4× extraction buffer to give a final concentration of 2×. Samples were subjected to 3–12% acrylamide BIS-TRIS native-PAGE (Life Technologies) and electrophoresed with anode buffer containing 50mM BIS-TRIS, 50mM tricine, and cathode buffer containing 50mM Bis-Tris, 50mM tricine, 0.004% CBB G-250 stain at 80V for an initial 1h and at 120V for the remaining time.

The BN-PAGE gels were directly incubated with 50mM HEPES-KOH, pH 7.5, 50mM G1P (Wako), 25mM AMP with or without Pho a (Sigma) at 30 °C for 16h with gentle shaking. The generated glucans were then stained with 1% iodine, 0.1% potassium iodine.

### Western blotting

Proteins were transferred to polyvinylidene fluoride (PVDF) membranes after SDS–PAGE, native-PAGE, or BN-PAGE. Membranes were treated as follows prior to blocking. (i) SDS–PAGE blots proceeded directly to the blocking step after transfer. (ii) Native-PAGE blots were fixed with 8% acetic acid for 10min and washed three times with water prior to the blocking procedure. (iii) BN-PAGE blots were washed with methanol prior to fixation with acetic acid and washing with water. The rest of the western blotting procedure was performed essentially as described by Crofts *et al.* (2012). Primary antibodies were used at the following dilutions: anti-SSI (Fujita *et al.*, 2006) at 1:1000, anti-SSIIa at 1:1000, anti-SSIIIa (Crofts *et al.*, 2012) at 1:1000, anti-SSIVb at 1:1000, anti-GBSSI (Fujita *et al.*, 2006) at 1:5000, anti-BEI (Nakamura *et al.*, 1992) at 1:2000, anti-BEIIa at 1:3000, anti-BEIIb (Nakamura *et al.*, 1992) at 1:3000, anti-PUL (Nakamura *et al.*, 1996) at 1:1000, anti-ISA1 at 1:1000, and anti-Pho1 (Satoh *et al.*, 2008) at 1:1000.

## Results

Attempts to purify reproducible quantities of amyloplasts from rice endosperm proved unsuccessful due to the large compound granules contained within the endosperm, and consequently whole-cell extracts were used as the starting material for all experiments.

### Expression and solubility of starch biosynthetic enzymes in developing rice endosperm

**Fig. 1.**
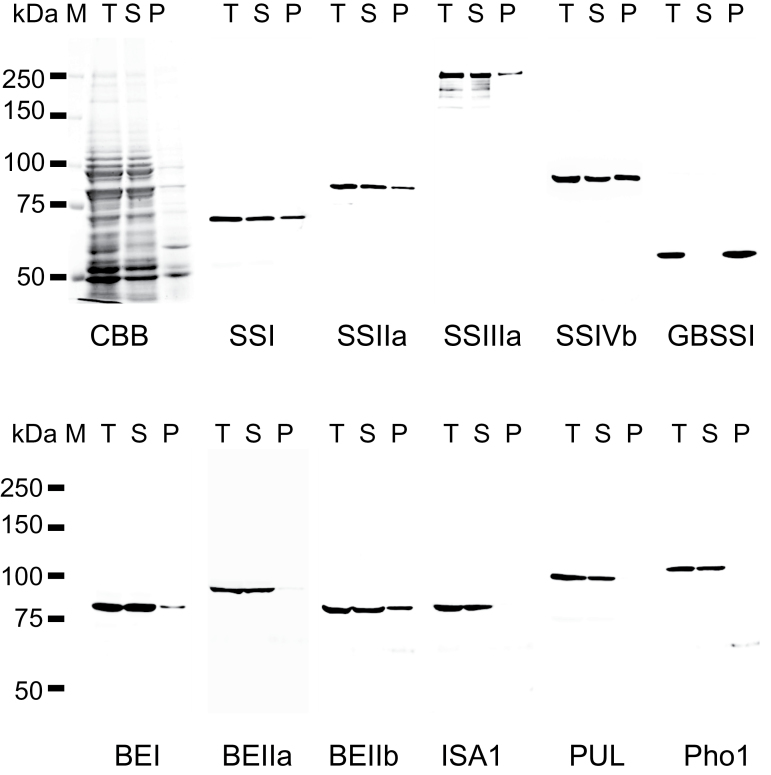
Expression and solubility of starch biosynthetic enzymes in rice endosperm, and confirmation of antibody specificity. Total (T), soluble (S), and insoluble, starch granule-associated (P) proteins were fractionated from rice developing endosperm and separated by SDS–PAGE. The gels were stained with Coomassie brilliant blue (CBB) or blotted onto membranes for western blotting using the antibodies indicated.

The expression and solubility of starch biosynthetic enzymes from rice developing endosperm (10–12 DAF) were analysed by western blotting (Fig. 1). Proteins were extracted using a denaturing buffer (see the Materials and methods) which also gelatinizes the starch to enable extraction of granule-bound proteins. Starch biosynthetic isozymes in developing rice endosperm (SSI, SSIIa, SSIIIa, SSIVb, GBSSI, BEI, BEIIa, BEIIb, ISA1, PUL, and Pho1; Hirose and Terao, 2004; Ohdan *et al.*, 2005) were analysed by western blotting (Fig. 1). The antibodies used for western blots were highly specific and were visualized as single bands, except for the anti-SSIIIa antibody which recognized multiple bands. However, the *ss3a* null mutants did not show any of the additional bands, suggesting that they represent truncated forms of SSIIIa (Supplementary Fig. S1 available at *JXB* online). Significant proportions of all the starch biosynthetic enzymes analysed here, except for GBSSI, were present in the soluble fraction. Currently it is unknown whether starch biosynthetic enzymes bound to the starch granule maintain their catalytic activities, except for GBSSI (Liu *et al.*, 2009*b*). The solubility of the starch biosynthetic enzymes was consistent among the different extraction buffers used for the study (Supplementary Fig. S2) and were used for analyses of protein–protein interactions.

### Elution of rice endosperm starch biosynthetic enzymes following gel permeation chromatography

**Fig. 2.**
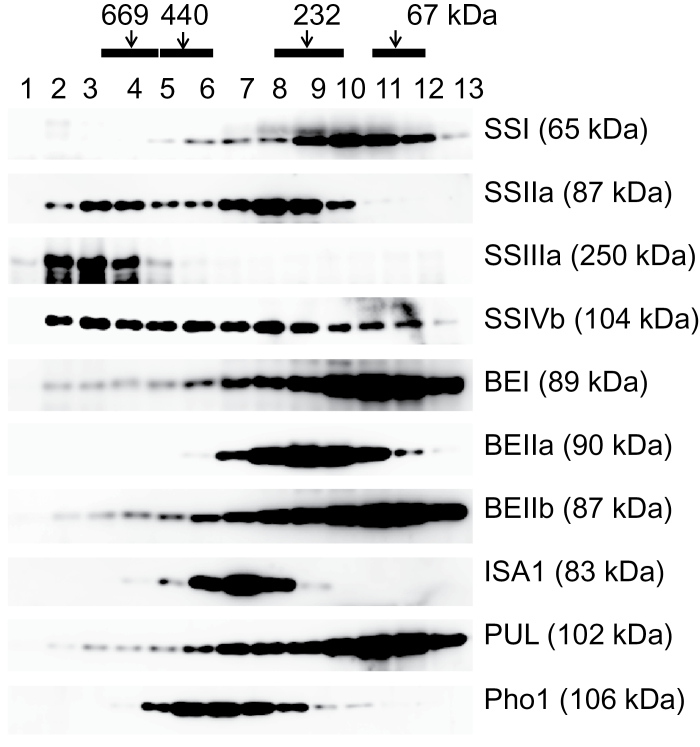
Molecular weight distributions of starch biosynthetic enzymes from developing rice endosperm determined by gel permeation chromatography. Soluble proteins from rice endosperm were separated on Superdex 200 and fractions analysed by western blotting using the antibodies indicated. The molecular weight of protein standards is shown at the top (black bars). Monomeric molecular weights of each isozyme are indicated on the right.

Soluble extracts from developing seeds were fractionated using a Superdex 200 gel filtration column. Native molecular weight standards were clearly separated by GPC (Fig. 2, black bars). Figure 2 shows that while some proteins could be detected at their expected monomeric size, all starch biosynthetic proteins analysed were eluted at higher molecular weights, consistent with the possibility that they may form higher order complexes.

Following GPC, SSI was eluted in higher molecular weight fractions (200–600kDa; fractions 5–9), in addition to its expected monomeric size (65kDa; fractions 10–13). SSIIa was eluted as two distinct peaks; between 150kDa and 400kDa (fractions 6–10) and also at >700kDa (fractions 2–5), but not at its expected monomeric size (87kDa). The majority of SSIIIa was eluted at >700kDa (fractions 1–5). In addition to its monomeric size of ~100kDa (fractions 10–12), SSIVb eluted between 200kDa and >700kDa (fractions 2–9). The majority of BEIIa eluted in fractions predicted to be <300kDa (fractions 7–11), whereas BEI and BEIIb showed a broad elution pattern ranging from >700kDa to their respective monomeric sizes (89kDa and 87kDa, respectively). ISA1 eluted between 200kDa and 400kDa (fractions 5-9), which was consistent with earlier observations that ISA1 forms homo-oligomers and hetero-oligomers with ISA2 (Utsumi *et al.*, 2006, 2011). PUL showed a similar broad distribution pattern (fractions 2–9) to BEI and BEIIb, in addition to eluting at its corresponding monomeric size of 102kDa (fractions 10–13). Pho1 was eluted between 100kDa and 700kDa (fractions 5–10) although recombinant rice Pho1 forms a dimer (Hwang *et al.*, 2010).

**Fig. 3.**
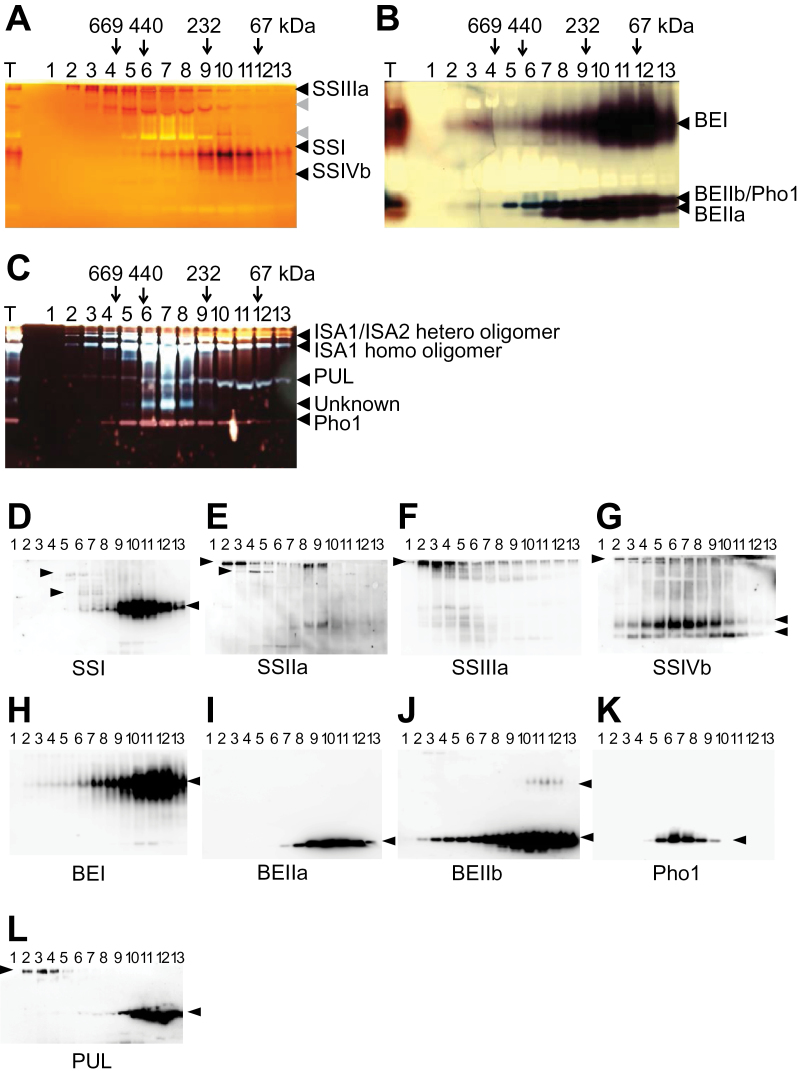
Starch biosynthetic enzyme activities analysed on non-denaturing zymograms and by western blotting following GPC of rice developing endosperm. Numbers at the top indicate the molecular weight of protein standards in kiloDaltons. (A) SS activity. (B) BE activity. (C) DBE activity. (D–G) Western blots of identical native-PAGE used for (A) were probed with SSI, SSIIa, SSIIIa, and SSIVb antibodies, respectively. (H–K) Western blots of identical native-PAGE used for (B) were probed with BEI, BEIIa, BEIIb, and Pho1 antibodies, respectively. (L) Western blots of identical native-PAGE used for (C) were probed with PUL antibody. Black arrowheads in (A–C) indicate activities of isozymes. Grey arrowheads in (A) indicate glycosyl hydrolase or glucan transferase activities. Arrowheads in (D–L) indicate polypeptides recognized by the antibody.

The same GPC fractions were analysed by native-PAGE/activity staining (Fig. 3A–C). The fractions with the highest enzymatic activity (Fig. 3) generally correlated with the strongest signals obtained by SDS–PAGE and western blotting (Fig. 2).

The activities of SSIIa and SSIVb could not be detected by non-denaturing, native-PAGE since japonica rice possesses inactive SSIIa (Nakamura *et al.*, 2005) and the activity of SSIVb was not high enough to detect with this assay (Y. Toyosawa *et al*., unpublished). SSI and SSIIIa activities were visualized using non-denaturing gels containing oyster glycogen as a primer, and incubated with 1mM ADP-glucose (Fig. 3A). SSI activity was found in high molecular weight fractions (fractions 5–9) as well as at its monomeric size (fractions 10–13). In contrast to SSI, the majority of SSIIIa activity was found in high molecular weight protein complexes >700kDa (peak activities were in fractions 2–5). The ‘starch synthase’ bands indicated in Fig. 3A with grey arrowheads are likely to be the outcome of either glycosyl hydrolase or glucan transferase activities since those bands were present in the absence of ADP-glucose (result not shown). Removal of short branches by these enzymes probably resulted in the production of linear chains which can bind to iodine.

Corresponding SS-native-PAGE gels were prepared and analysed by western blotting (Fig. 3D–G). The results suggested that the faint SS activity below the SSI was SSIVb since the immune-detected protein was coincident with SS activity (Fig. 3A). Furthermore, western blotting of native-PAGE gels revealed that SSIIa and SSIVb in fractions 2 and 3 were present near the top of the gels, possibly as components of protein complexes, given that they were detected in fractions corresponding to molecular weight >700kDa.

The activities of BE isozymes were visualized by zymogram analysis in the presence of G1P as a substrate based on the Pho a stimulation assay (Fig. 3B). The strongest BEI activity was found in fractions 10–12 which contained the largest amounts of BEI protein (Figs 2, 3H). BEI activity was also observed in fractions 2–9, corresponding to BEI protein between 200 kDa and >700kDa (Figs 2, 3H). BEIIb and Pho1 co-migrate on native-PAGE as described by Yamanouchi and Nakamura (1992). It was also confirmed, by western blotting of the corresponding native-PAGE, that BEIIb and Pho1 migrated to the same position (Fig. 3J, K). BEIIb activities were detected in fractions 2–13 and the highest activities were found in fractions 8–12 (purple). The fractions with high Pho1 protein content exhibited blue activity bands (fractions 5–7) indicative of glucan elongation. BEIIa peak activities were found in fractions 6–13, and the molecular weight distribution of BEIIa was narrower than that or BEIIb or BEI (Figs 2, 3B, I). DBE and Pho1 activity bands were visualized by native-PAGE which contains potato amylopectin as a substrate (Fig. 3C). There were at least three ISA activity bands which peaked in fractions 6–8 corresponding to ISA1/ISA2 hetero-oligomers and ISA1 homo-oligomers (Fig. 3C) as previously described by Utsumi *et al.* (2011). Hydrolytic activity of PUL found in fractions 2–13 (Fig. 3C) correlated with the amount of PUL protein (Fig. 2). In addition, western blotting of identical native-PAGE gels with anti-PUL revealed that significant amounts of PUL in fractions 2–4 were present near the top of the gel in high molecular weight fractions (Fig. 3L). Strong Pho1 activity was seen in fractions 5–9. A hydrolytic activity (marked ‘unknown’) in fractions 6–8 was not recognized by ISA, PUL, or Pho1 antibodies (data not shown).

### Analyses of starch biosynthetic protein complexes in rice endosperm by immunoprecipitation

**Fig. 4.**
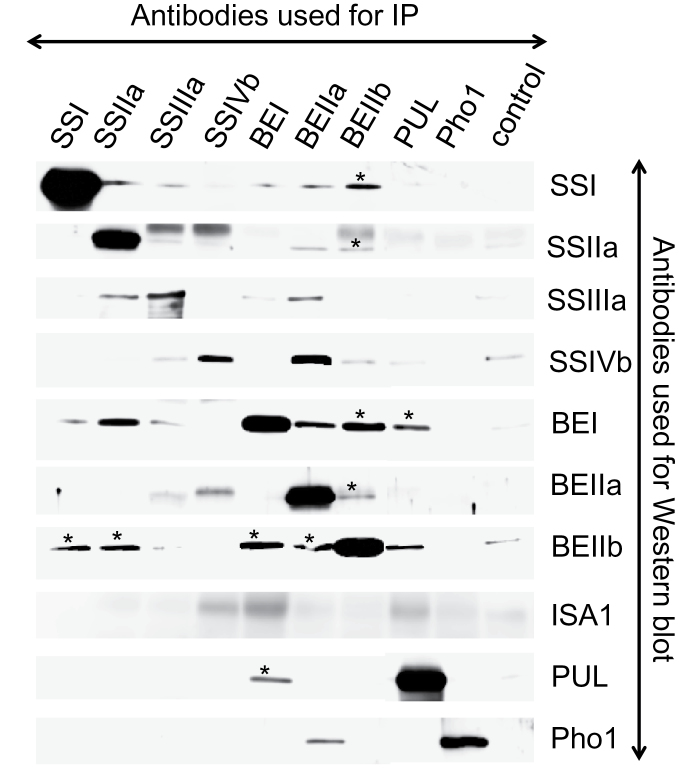
Analyses of protein–protein interaction between rice starch biosynthetic isozymes by co-immunoprecipitation (IP). Soluble proteins from rice developing endosperm were immunoprecipitated using antibodies as described. Pre-immune serum was used as a control. The antibodies used for western blots are indicated on the right. Asterisks indicate the interactions confirmed by reciprocal IP.

In order to investigate possible interacting partners among starch biosynthetic isozymes, immunoprecipitation was carried out using soluble rice endosperm extract and isozyme-specific antibodies or a pre-immune serum control (Fig. 4, and summarized in Table 2). Each antibody recognized and could immunoprecipitate its respective antigen, except for anti-ISA1 antibodies which could not immunoprecipitate the native protein (results not shown).

Strong, pairwise, associations obtained by reciprocal co-immunoprecipitation were observed for SSI–BEIIb, SSIIa–BEIIb, BEI–BEIIb, BEI–PUL, and BEIIa–BEIIb. Clear, but less intense signals were obtained from reciprocal co-immunoprecipitation experiments for the pairwise interactions SSI–BEI, SSIIa–SSIIIa, SSIIIa–BEI, and SSIVb–BEIIa.

In some instances, clear western blot signals were obtained from only one side of the co-immunoprecipitation, including SSIIa–BEI, BEIIa–BEI, BEIIa–Pho1, and PUL–BEIIb (first acronym, antibody used for immunoprecipitation; second acronym, isozyme detected by western blotting), whereas the reciprocal co-immunoprecipitation did not show the same interaction. Similarly, relatively weaker (but clear) immunodetection of co-precipitated protein was observed for SSIIa–SSI, BEIIa–SSI, SSIIa–BEI, BEIIa–SSIIa, BEIIa–SSIIIa, and BEIIa–BEI, but not in the reciprocal direction.

### Activity analyses of branching enzyme complexes from rice endosperm using BN-PAGE

Individual activities of most starch biosynthetic enzymes were observed in the GPC high molecular weight fractions by native-PAGE as shown in Fig. 3. However, whether the starch biosynthetic protein complexes possess catalytic activity cannot be presumed. BN-PAGE was therefore performed since this technique maintains the interactions of protein complexes, reflects the molecular weight of the protein complexes, and the influence of differences in isoelectric point of individual isozymes is minimal (Eubel *et al.*, 2005). The use of gradient gels for BN-PAGE also gave better resolution of high molecular weight protein complexes compared with GPC.

**Fig. 5.**
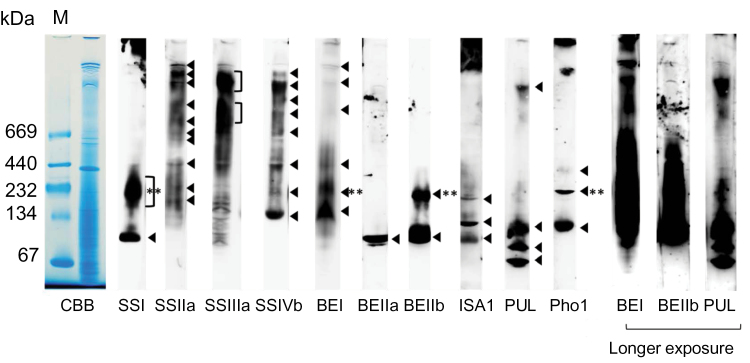
Western blots of BN-PAGE shows the formation of starch biosynthetic enzyme complexes from developing rice endosperm. Arrowheads and brackets indicate the presence of polypeptides recognized by antibodies. Double asterisks indicate the co-migrating enzymes. (This figure is available in colour at *JXB* online.)

The presence of starch biosynthetic enzymes in high molecular weight complexes was confirmed by western blotting of a BN-PAGE gel (Fig. 5) and two-dimensional gels in which BN-PAGE and SDS–PAGE were used for the first and second dimension, respectively (Supplementary Fig. S3 at *JXB* online). Western blotting of BN-PAGE (Fig. 5) was performed using slices of lanes from a single gel so the molecular weight of proteins is directly comparable. Western blotting of two dimensional gels (Supplementary Fig. S3) showed that the enzymes present at high molecular weight on BN-PAGE (Fig. 5) were present as protein complexes since they migrated to their expected monomeric molecular weight in two-dimensional gels.

SSI, BEIIb, and Pho1 co-migrated to a position below the 232kDa molecular weight standard in addition to their respective monomeric protein sizes. SSIIa, SSIIIa, and SSIVb migrated to positions corresponding to multiple, different, molecular weights, and significant proportions of these enzymes were found in complexes >700kDa. While most of the BEIIa was present at its monomeric molecular weight, BEI was present over a broad range of molecular sizes. BEI, BEIIb, and PUL were also present over a broad range of molecular sizes (Fig. 5, longer exposure). The data are consistent with the molecular weight distributions of starch biosynthetic isozymes observed by GPC (Fig. 2).

**Fig. 6.**
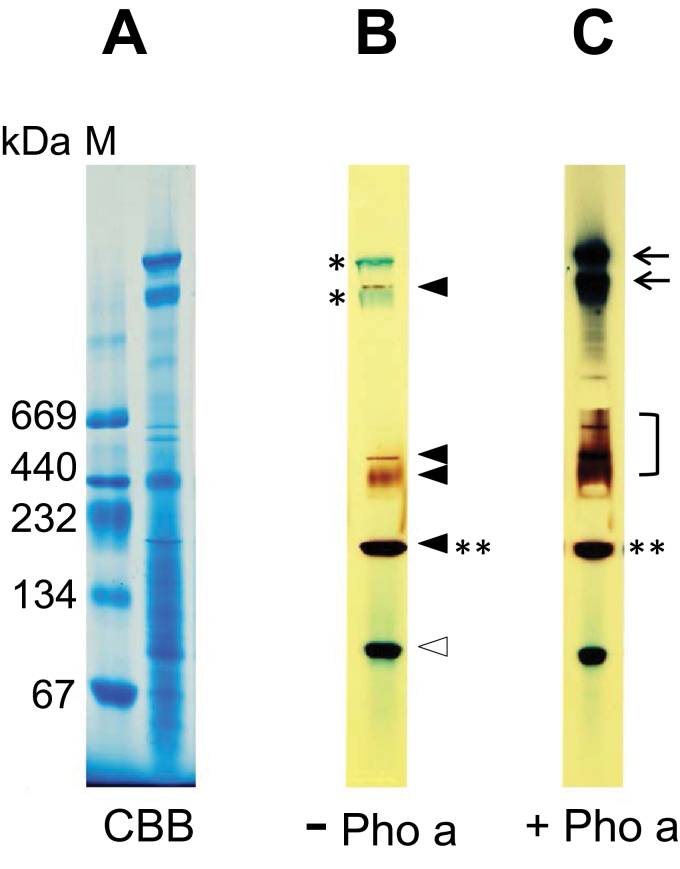
BN-PAGE activity staining shows the glucan synthesis activities of starch biosynthetic enzyme complexes from developing rice endosperm. (A) CBB-stained BN-PAGE gel showing separation of protein complexes. (B) Synthesis of glucan by endogenous BE–Pho1 interaction. The BN-PAGE gel was incubated with 50mM G1P, and the generated glucans were visualized by iodine staining. (C) Stimulation of glucan synthesis by exogenous phosphorylase a (Pho a). The BN-PAGE gel was incubated with 50mM G1P and rabbit Pho a, and stained with iodine. Black arrowheads indicate glucan synthesis activity by interaction of endogenous BEs and Pho1. The white arrowhead indicates glucan synthesis arising from co-migration of BEs and Pho1 due to their similar monomeric sizes. Arrows and the bracket indicate the stimulation of glucan synthesis by addition of exogenous Pho a. Single asterisks indicate residual CBB from the BN-PAGE running buffer (not glucans stained by iodine). The activity band indicated with double asterisks corresponds to SSI, BEI, BEIIb, and Pho1as indicated in Fig. 5

Incubation of the BN-PAGE gel (Fig. 6A; CBB stained) with G1P followed by iodine staining demonstrated α-glucan synthesis in a wide molecular weight range of protein complexes, at ~100, 200, 440, 500, and 1000kDa (Fig. 6B). The 100kDa glucan band was likely to be generated by co-migration of monomeric Pho1 and BEs (BEIIa and BEIIb) as observed by western blotting of the BN-PAGE gel (Fig. 5; Supplementary Fig. S4 at *JXB* online). Addition of exogenous Pho a to the G1P-containing reaction mixture led to a significant increase in amounts of generated α-glucans at ~400–600kDa and ~1000kDa. A very discrete brown band at ~670kDa and the two, nearby, lower bands became apparent (Fig. 6C), indicating the presence of BE activities which are able to interact with the exogenous Pho a.

The colour difference of iodine staining generally reflects the structure of α-glucan (Bailey and Whelan, 1961). The α-glucans stained in brown contain more branched and shorter glucans, while those stained in dark blue have less branched and longer glucan structures (Guan and Preiss, 1993). The 400–700kDa glucan bands were brown while two bands at ~1000kDa were dark blue. This indicates that the activities of BE isozymes are relatively higher in 400–700kDa bands than in the ~1000kDa bands (Fig. 6C).

It is possible that short and undetectable amounts of α-glucan were present within the starch biosynthetic enzyme complexes analysed in Fig. 6. However, incubation of the BN-PAGE with G1P did not show any iodine-stained glucans (result not shown). Therefore, the generated α-glucans in Fig. 6 are proposed to be generated by the co-ordinated actions of Pho1 and BEs.

## Discussion

Interactions between starch biosynthetic isozymes have been demonstrated previously in wheat, maize, and barley developing endosperm (Tetlow *et al.*, 2004, 2008; Hennen-Bierwagen *et al.*, 2008, 2009, Liu *et al.*, 2009*a*; Ahmed *et al.*, 2015). Since the gene structures and functions of starch biosynthetic enzymes are well conserved among cereals (Fujita and Nakamura, 2012), it was hypothesized that starch biosynthetic isozymes from rice endosperm interact with each other to form functional protein complexes. The present study is the first demonstration of active, high molecular weight, starch biosynthetic enzyme complexes in rice endosperm. These discoveries were made possible with the use of a comprehensive collection of isozyme-specific antibodies. The similarities and differences in protein complex formation among wild-type wheat (Tetlow *et al.*, 2008), maize (Tetlow *et al.*, 2004; Hennen-Bierwagen *et al.*, 2008, 2009; Liu *et al.*, 2009*a*), and rice are summarized in Tables 1 and 2, although the possibility remains that the differences may arise not only from each plant-specific unique function, but also from the difference in experimental approaches such as choice of endosperm developmental stage and whether amyloplasts or whole-cell extracts are used as the starting material.

**Table 1.** Molecular weight distribution patterns for starch biosynthetic enzymes determined by GPC in wheat, maize, and rice

Isozyme	Species	Molecular weight (kDa)				
		<700	400–600	200–400	100–200	>100
SSI	Wheat^*a*^	ND	–	+ *	+*	+++*
	Maize^*b*^	–	+	++	++	+
	Rice ^c^	–	**+**	**+**	**+**	**+++**
SSIIa	Wheat^*a*^	ND	–	+ *	+ *	++*
	Maize^*b*, *d*, *e*^	–	–	+++	+	++
	Rice^*c*^	++	+	+++	–	–
SSIIIa	Wheat	ND	ND	ND	ND	ND
	Maize^*d*, *e*^	++	+	–	–	–
	Rice ^c^	**+++**	**++**	+	–	–
SSIVb	Wheat	ND	ND	ND	ND	ND
	Maize	ND	ND	ND	ND	ND
	Rice^*c*^	++	**++**	**++**	**+**	–
BEI	Wheat^*a*^	ND	–	–	–	–
	Maize^*b*, *d*, *e*^	–	–	–	–	+++
	Rice^*c*^	**+**	**+**	**+**	**++**	**+++**
BEIIa	Wheat^*a*^	ND	+**	++**	+**	++**
	Maize^*d*, *e*^	+	+	+	++	+++
	Rice^*c*^	–	–	**+**	**+++**	**+++**
BEIIb	Wheat^*a*^	ND	–	++ **	–	++ **
	Maize^*d*, *e*^	+	+	++	+++	+++
	Rice^*c*^	**+**	**+**	**++**	**++**	**++**
ISA1	Wheat	ND	ND	ND	ND	ND
	Maize^*f*^	–	–	+++	+	–
	Rice^*c*^	–	–	**+++**	**++**	–
PUL	Wheat	ND	ND	ND	ND	ND
	Maize	ND	ND	ND	ND	ND
	Rice^*c*^	+	**+**	**++**	**++**	**+++**
Pho1	Wheat	ND	ND	ND	ND	ND
	Maize^*b*^	–	–	+++	+	–
	Rice^*c*^	–	**+**	**+++**	**+**	**–**

-, No western blot signals; +, less than 20% of total western blot signal; ++, 20–50% of total western blot signal; +++, more than 50% of total western blot signal. Bold character indicates that the isozymes in those fractions were active. ND, not determined.

*Sum of SS isozyme activities including SSI and/or SSIIa; **sum of BE isozyme activities including BEIIa and/or BEIIb.

^*a*^ Tetlow *et al.* (2008).

^*b*^ Hennen-Bierwagen *et al.* (2008).

^*c*^ Derived from Figs 2 and 3 of this study.

^d^ Liu *et al.* (2009*a*).

^*e*^ Hennen-Bierwagen *et al.* (2009).

^*f*^ Kubo *et al*. (2010).

**Table 2.** Comparison of protein–protein interactions among starch biosynthetic isozymes in wheat, maize, and rice determined by co-immunoprecipitation

	Reciprocal		One sided	
	Strong signal	Weak signal	Strong signal	Weak signal
Wheat	***BEI-BEIIb*** ^*a*^		Pho1–BEI^*a*^	
	***SSI-BEIIb*** ^*b*^		Pho1–BEIIb^*a*^	
	***SSII-BEIIb*** ^*b*^		**BEIIa-SSI** ^*b*^	
			**BEIIa-SSII^b^	
Maize	*SSI-SSIIa^*c*, *e*, *f*, *g*^	SSIII–PPDK^*d*^	**BEI-BEIIb** ^*e*^	
	***SSI-BEIIa*** ^*c*^		*SSIII-SSIIa ^*d*^	
	**SSI-BEIIb** ^*c*, f, g^		*SSIII-BEIIa ^*d*^	
	**SSIIa-BEIIb** ^*c*, *e*, *f*, *g*^		SSIII–BEIIb^*d*^	
Rice^*h*^	**SSI-BEIIb**	SSI–BEI	SSIIa–BEI	*SSIIa-SSI
	**SSIIa-BEIIb**	*SSIIa-SSIIIa	BEIIa–BEI	**BEIIa-SSI**
	**BEI-BEIIb**	SSIIIa–BEI	BEIIa–Pho1	SSIIa–BEI
	BEI–PUL	SSIVb–BEIIa	PUL–BEIIb	**BEIIa-SSIIa
	BEIIa–BEIIb			BEIIa–SSIIIa
				BEIIa–BEI

The column heading ‘one sided’ indicates the antibody used for immunoprecipitation on the left and the co-precipitated isozymes detected by western blotting on the right.

The interactions common among wheat, maize, and rice are indicated in bold. The interactions common between maize and rice are indicated in with *. The interactions common between wheat and rice are indicated in with **.

^*a*^ Tetlow *et al.* (2004).

^*b*^ Tetlow *et al.* (2008).

^*c*^ Hennen-Bierwagen *et al.* (2008).

^*d*^ Hennen-Bierwagen *et al.* (2009).

^*e*^ Liu *et al.* (2009).

^f^ Liu *et al*. (2012*a*).

^*g*^ Liu *et al*. (2012*b*).

^*h*^ Figure 4 of this study.

Abbreviations: BE, branching enzyme; BN-PAGE, blue native polyacrylamide gel electrophoresis; CBB, Coomassie brilliant blue; DAF, days after flowering; DBE, debranching enzyme, G1P, glucose 1-phosphate; GBSSI, granule-bound starch synthase I; GPC, gel permeation chromatography; HPLC, high-performance liquid chromatography; ISA; isoamylase; KLH, keyhole limpet haemocyanin; Pho, phosphorylase; PUL, pullulanase; PVDF, polyvinylidene fluoride; SS, starch synthase.

### Comparisons of molecular weight distribution patterns of protein complexes among wheat, maize, and rice starch biosynthetic enzymes by gel filtration chromatography

The elution patterns of rice starch biosynthetic enzymes were classified into two major groups (Fig. 2). The enzymes eluted in a broad molecular weight range, and those speculated to form multiple, differently sized, protein complexes were SSI, SSIIa, SSIVb, BEI, BEIIb, and PUL (Fig. 2). The enzymes which eluted in narrow molecular weight ranges were SSIIIa, BEIIa, ISA1, and Pho1 (Fig. 2).

The monomeric sizes of starch biosynthetic isozymes are similar among the monocot species studied to date. In wheat, SSI, SSIIa, and BEIIb were present at their monomeric size in early stages of seed development, while the formation of the heterotrimer was more pronounced at mid-development (Tetlow *et al.*, 2008). GPC elution patterns of maize SSI (Liu *et al.*, 2009*a*) and SSIIa (Hennen-Bierwagen *et al.*, 2008, 2009; Liu *et al.*, 2009*a*) were similar to those described for wheat, suggesting that they are also part of a trimer with SBEIIb, as well as higher order multimers involving SSIII which were eluted at ~670kDa (Hennen-Bierwagen *et al.*, 2008, 2009). In wild-type maize, BEI was detected only as a monomer although in *ae* ^*–*^ mutants, lacking BEIIb, BEI was shown to interact with SSI, SSII, and starch phosphorylase (Hennen-Bierwagen *et al.*, 2008; Liu *et al.*, 2009*a*). In wheat, BEI interacts with BEIIb and starch phosphorylase (Tetlow *et al.*, 2004). In rice, the majority of BEIIa appeared to be monomeric, whereas BEIIb was eluted in a broad range of fractions from the monomeric size to >700kDa, similar to maize BEIIb (Hennen-Bierwagen *et al.*, 2008, 2009). The elution pattern of rice Pho1 was similar to that of maize, which is predicted to form homotetramers and hetero-oligomers (Ahmed *et al.*, 2015)

Following GPC, most starch biosynthetic isozymes from rice endosperm were at least partially eluted in the high molecular weight fraction. SSIIa, SSIIIa, SSIVb, BEI, BEIIb, and PUL were co-eluted in fractions corresponding to a mass >700kDa (Fig. 2), consistent with the hypothesis that they are components of one or more multienzyme complexes. The elution pattern of rice SSIIa was distinct from that observed in maize and wheat in that it was not found in its monomeric size at all. This is interesting because SSIIa is known to be inactive in japonica rice varieties (Nakamura *et al.*, 2005), but may still be of importance for formation of protein complexes, since evidence from maize demonstrated that it forms the core of a trimeric complex with SSI and BEIIb (Liu *et al*., 2012*b*).

### Comparison of protein–protein interactions among wheat, maize, and rice starch biosynthetic enzymes

The results of co-immunoprecipitation experiments indicate that the pairwise interactions SSI–SSIIa, SSI–BEIIa, SSI–BEIIb, SSIIa–BEIIb, and BEI–BEIIb are common among wheat, maize, and rice (Table 2). Further interacting isozymes found in common between maize and rice, but not investigated in wheat, were SSIIa–SSIIIa, SSIIIa–BEII, and SSIIIa–BEIIb.

The finding of an interaction between branching and de-branching enzymes (BEI–PUL) in rice by immunoprecipitation was unexpected, but is supported by the similar elution patterns shown by GPC (Fig. 2). Although there is no other evidence showing interaction of PUL and BEs, it is worth noting that the *pul* ^*–*^ null mutant in maize (*zpu1-204*) showed a reduction in BEI activity during germination, and BEIIa activity in leaf and developing seeds (Dinges *et al.*, 2003), and rice *pul* ^*–*^ mutants exhibited a slight decrease in BEIIb activity (Fujita *et al.*, 2009) suggesting some functional and/or physical interaction between these two classes of enzyme. A detailed analysis of the relationship between PUL and BEs in starch synthesis and degradation will be the subject of future investigations.

**Fig. 7.**
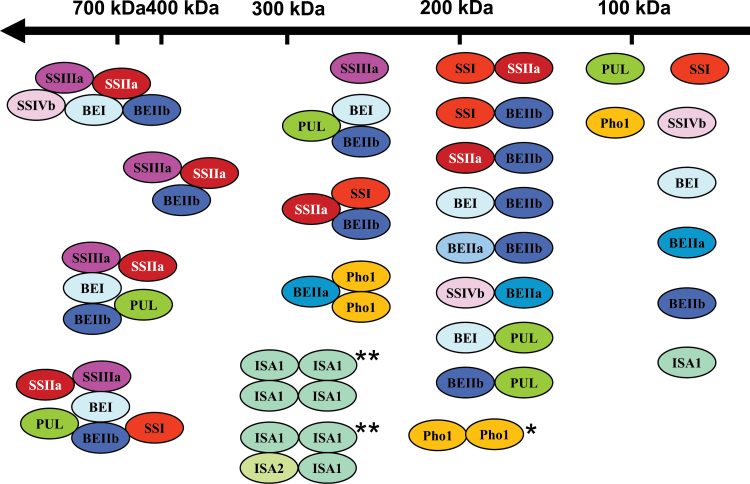
Possible protein–protein interactions in rice developing endosperm. Potential protein–protein interactions among starch biosynthetic enzymes of developing rice endosperm were deduced from western blotting (Fig. 2) and native-PAGE zymograms (Fig. 3) following GPC, co-immunoprecipitation experiments (Fig. 4), and BN-PAGE (Figs 5, 6) performed in this study. SS isozymes are in red, BE isozymes are in blue, DBE isozymes are in green, and Pho1 is in yellow. SSIIa is inactive in japonica rice (therefore not detected in Fig. 3) and indicated with white font. Single and double asterisks indicate formation of Pho1 dimers and ISA homo-oligomers confirmed in this study, previously reported by Hwang *et al.* (2010) and Utsumi *et al.* (2006), respectively. Other SS, BE, and DBE oligomers may occur, but are not included in this figure. The stoichiometric relationships between isozymes in high molecular weight complexes are unknown.

Based on GPC, co-immunoprecipitation, and BN-PAGE, possible combinations of protein–protein interactions in developing rice endosperm are postulated in Fig. 7. All of these protein complexes, in addition to the monomeric isozymes, may co-exist within the same cell in developing rice endosperm. The various dimeric interactions estimated at ~200kDa may be constituents of the larger protein complexes observed at 700kDa estimated to consist of SSIIa–SSIIIa–SSIVb–BEI, SSIIa–SSIIIa–BEIIb, SSIIa–SSIIIa–BEI–BEIIb–PUL, and SSI–SSIIa–SSIIIa–BEI–BEIIb–PUL. SSIIIa and BEIIa showed some interaction based on co-immunoprecipitation analyses, but the interaction was not detectable by western blotting after GPC or BN-PAGE. This suggests that the relative amounts of SSIIIa and BEIIb in this complex were low or possibly that the complex turns over very quickly and is lost during further electrophoretic and chromatographic separation.

The stoichiometry of each isozyme present in such complexes is currently under investigation. The protein–protein interactions involving SSIII in maize endosperm were proposed to be involved in co-ordination of the interactions among plastidal starch biosynthetic enzymes including SSIIa, BEIIa, and BEIIb, suggesting a regulatory function for SSIII in carbon partitioning (Hennen-Bierwagen *et al.*, 2009).

The formation of homo-oligomers by both Pho1 and ISA1 confirms previous studies (Hwang *et al.*, 2010; Utsumi *et al.*, 2011). ISA1 was previously determined to form homohexamers (~530kDa) and hetero-oligomers with five ISA1 and one ISA2 (~450kDa) (Utsumi *et al.*, 2006); however, a recent crystal structure analysis of *Chlamydomonas* ISA1 revealed that ISA1 has an elongated structure and forms an end-to-end dimer (Sim *et al.*, 2014). Therefore, the actual molecular weight of rice ISA1 oligomer may be smaller than its apparent molecular weight as determined by GPC.

The possibility that SSs, BEs, and DBEs also form oligomers cannot be ruled out. Interactions between Pho1 and BEI or BEIIb were not observed by co-immunoprecipitation (Fig. 4), but their interactions were strongly suggested by BN-PAGE (Figs 5, 6). One of the reasons for this may be due to the low abundance of specific complexes or the weakness of the interactions. Further approaches, including chemical cross-linking and/or enrichment of target complexes, will be required to resolve such questions.

### Significance of physical interactions between SSI and BEIIb in rice endosperm

The interaction of rice SSI and BEIIb was demonstrated by GPC (Figs 2, 3), co-immunoprecipitation, and BN-PAGE (Fig. 5). Consistent with this, functional interactions between recombinant rice SSI and BEI, BEIIa, or BEIIb were shown, and citrate-dependent SSI efficiently synthesizes glucans in the presence of any one of the BE isozymes even in the absence of glucan primers (Nakamura *et al.*, 2014).

The current consensus is that SSI primarily elongates the glucan branches with degree of polymerization (DP) 6–7 which are generated by BEIIb (Nakamura *et al.*, 2010, 2014; Abe *et al.*, 2014). Rice plants lacking both SSI and BEIIb were sterile, whereas rice plants lacking SSI and BEI remain fertile (Abe *et al.*, 2014). This suggests that the presence of both SSI and BEIIb is crucial in formation of amylopectin and cannot be totally compensated by BEI or BEIIa isozymes. However, the interaction between SSI and BEI is likely to be complemented/compensated by other BE isozymes such as BEIIa and/or BEIIb. In fact, mutant rice which lacks BEIIb exhibited reduced SSI activity associated with less soluble SSI protein, although the total amount of SSI was similar to that of the wild type (Nishi *et al.*, 2001; Abe *et al.*, 2014). Overexpression of BEIIb in rice resulted in the accumulation of water-soluble phytoglycogen (Tanaka *et al.*, 2004), indicating that the ratio of BEIIb and other starch biosynthetic enzymes such as SSI and DBEs may also be important.

### Issues and prospects in analysis of starch biosynthetic isozyme complexes in developing rice endosperm

The present study has shown that the soluble starch biosynthetic enzymes in developing rice endosperm form enzymatically active multiprotein complexes, several of which are common to wheat and maize. At the same time, significant differences in the combinations of isozyme interactions among cereals were also revealed, although there are some gaps in information among the species mainly due to the availability of specific antibodies.

Previous studies on wheat and maize have demonstrated that the formation of such complexes is catalysed as a result of post-translational phosphorylation of some enzymes. Investigation of this in rice could not be pursued as large amounts of intact amyloplasts could not be purified, due to the presence of multiple, compound starch granules in amyloplasts (Matsushima *et al.*, 2010), and whole-cell extracts would contain too many non-specific phosphatases as well as protein kinases from different subcellular compartments. Establishing a new method which facilitates isolation of intact amyloplasts from rice endosperm will require alternative approaches to those currently employed in other cereals.

The use of BN-PAGE for the analyses of starch biosynthetic enzyme complexes provided a new and useful perspective to the analysis of protein complex activities. The present study has shown that rice starch biosynthetic protein complexes generated glucans in the absence of any added primers in the BN-PAGE gels. Addition of glucan primers or substrates into BN-PAGE gels might facilitate detection of the synthesis and/or degradation of starch by protein complexes, separated according to their native molecular weight. In addition, western blot analysis of BN-PAGE gels (Fig. 5) showed that this technique can resolve the components of protein complexes of similar molecular weight. For example, SSI and BEIIb co-migrated to ~230kDa, and were clearly separated from the corresponding monomers on BN-PAGE (Fig. 5), while 230kDa complexes and monomeric proteins were eluted as a broad peak following GPC (Fig. 2). Specific areas of the BN-PAGE gel with starch synthesis activity will be excised and analysed by mass spectrometry in order to identify other components of the protein complex.

More detailed analysis of the glucan products generated by complexes, coupled with the use of rice mutants lacking specific starch biosynthetic isozyme(s), will facilitate a clearer understanding of the properties of these protein complexes. To date, demonstration of protein–protein interactions between starch biosynthetic enzymes has been confined to endosperm of cereal species. It will be important to determine whether these interactions are tissue specific or also occur in different organs such as leaves or roots. In order to understand whether protein complex formation was acquired through evolution to produce and store starch efficiently, it will also be of interest to examine species such as microalgae.

## Supplementary data

Supplementary data are available at *JXB* online.

Supplementary Materials and methods.

Figure S1. Specificity of SSIIIa antibody.

Figure S2. Comparison of starch biosynthetic enzymes extracted in different buffer solutions.

Figure S3. BN/SDS 2D-PAGE showing molecular weight distribution of native starch biosynthetic enzymes.

Supplementary Data
