# Supplementary material for: Amylopectin biosynthetic enzymes from developing rice seed form enzymatically active protein complexes
Source: J Exp Bot. 2015 May 15;66(15):4469–82. doi: 10.1093/jxb/erv212 (PMC4507757; doi:10.1093/jxb/erv212)
Supplement: Supplementary Data [file supp_66_15_4469__index.html]

Amylopectin biosynthetic enzymes from developing rice seed form enzymatically active protein complexes — Amylopectin biosynthetic enzymes from developing rice seed form enzymatically active protein complexes — Supplementary Data 

# Amylopectin biosynthetic enzymes from developing rice seed form enzymatically active protein complexes

## Supplementary Data

Data files

**Files in this Data Supplement:**

- Supplementary Data - Supplementary Data
