# Supplementary figures and images for: Amylopectin biosynthetic enzymes from developing rice seed form enzymatically active protein complexes
Source: J Exp Bot. 2015 May 15;66(15):4469–82. doi: 10.1093/jxb/erv212 (PMC4507757; doi:10.1093/jxb/erv212)

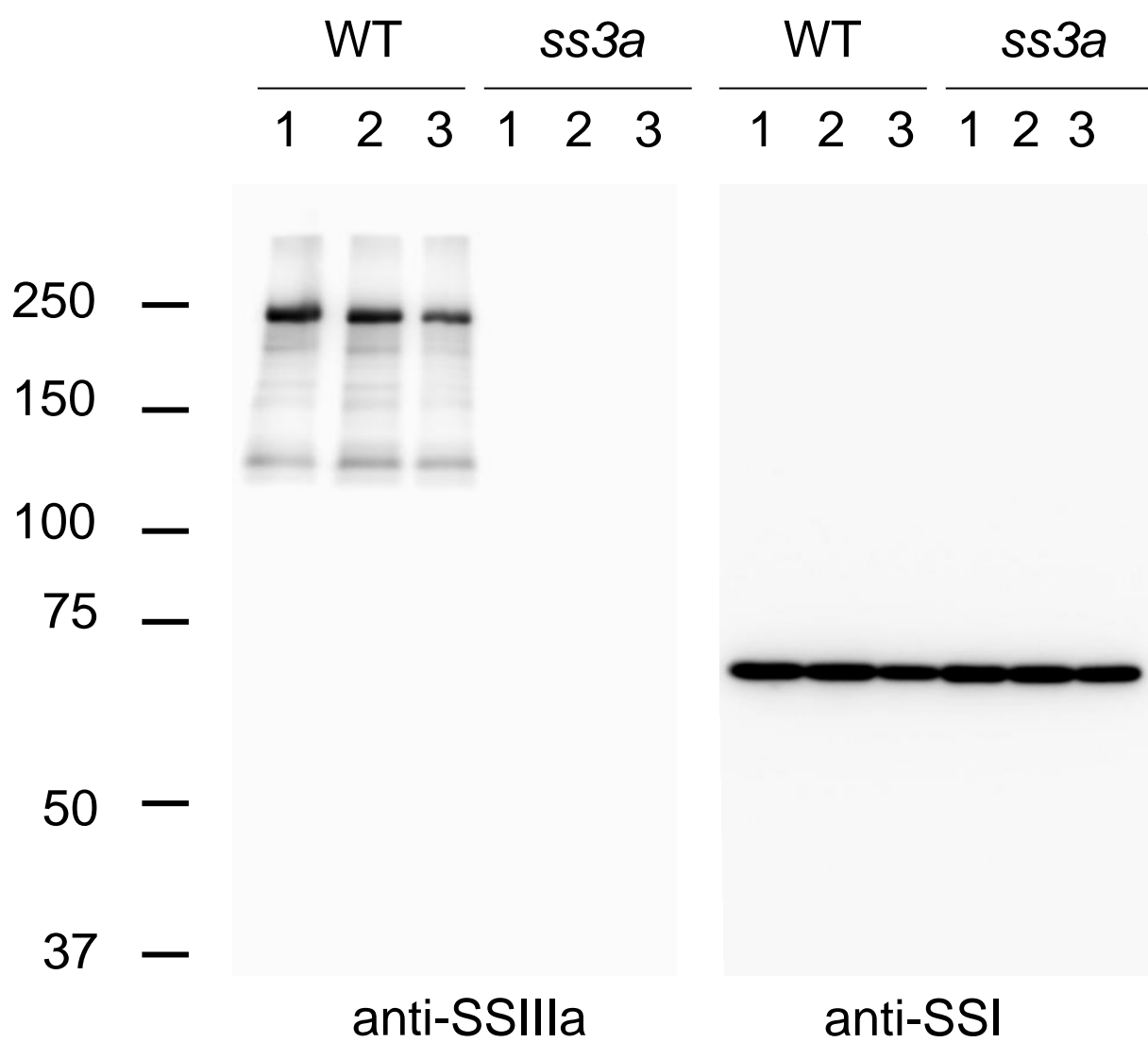

**Fig. S1**

**A**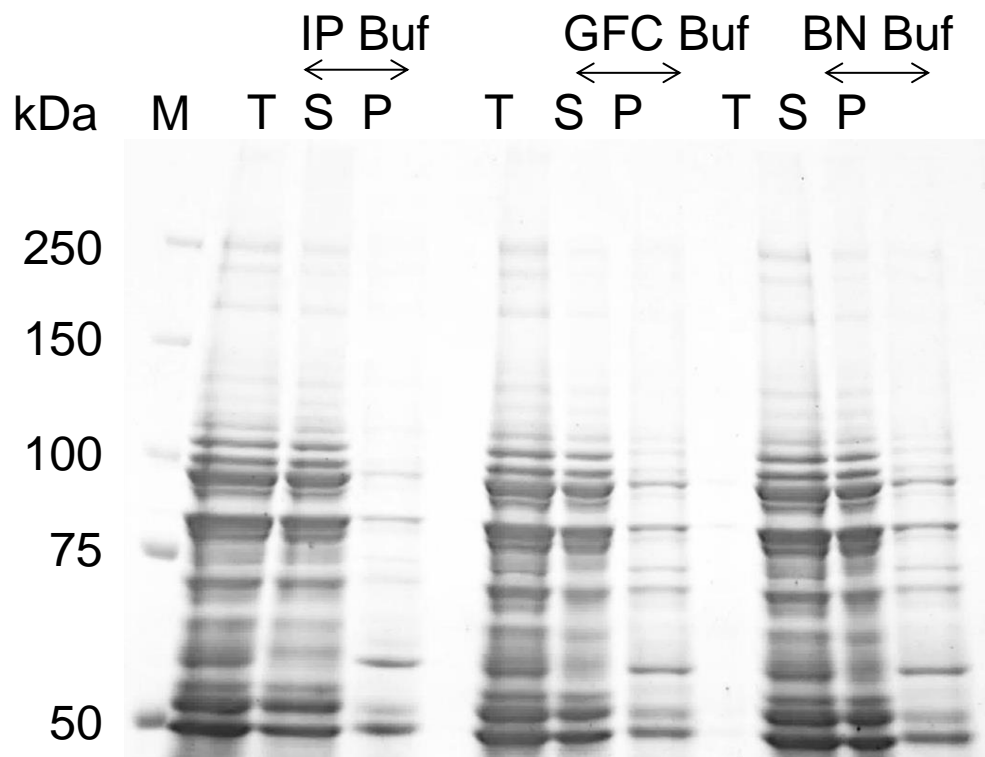**B**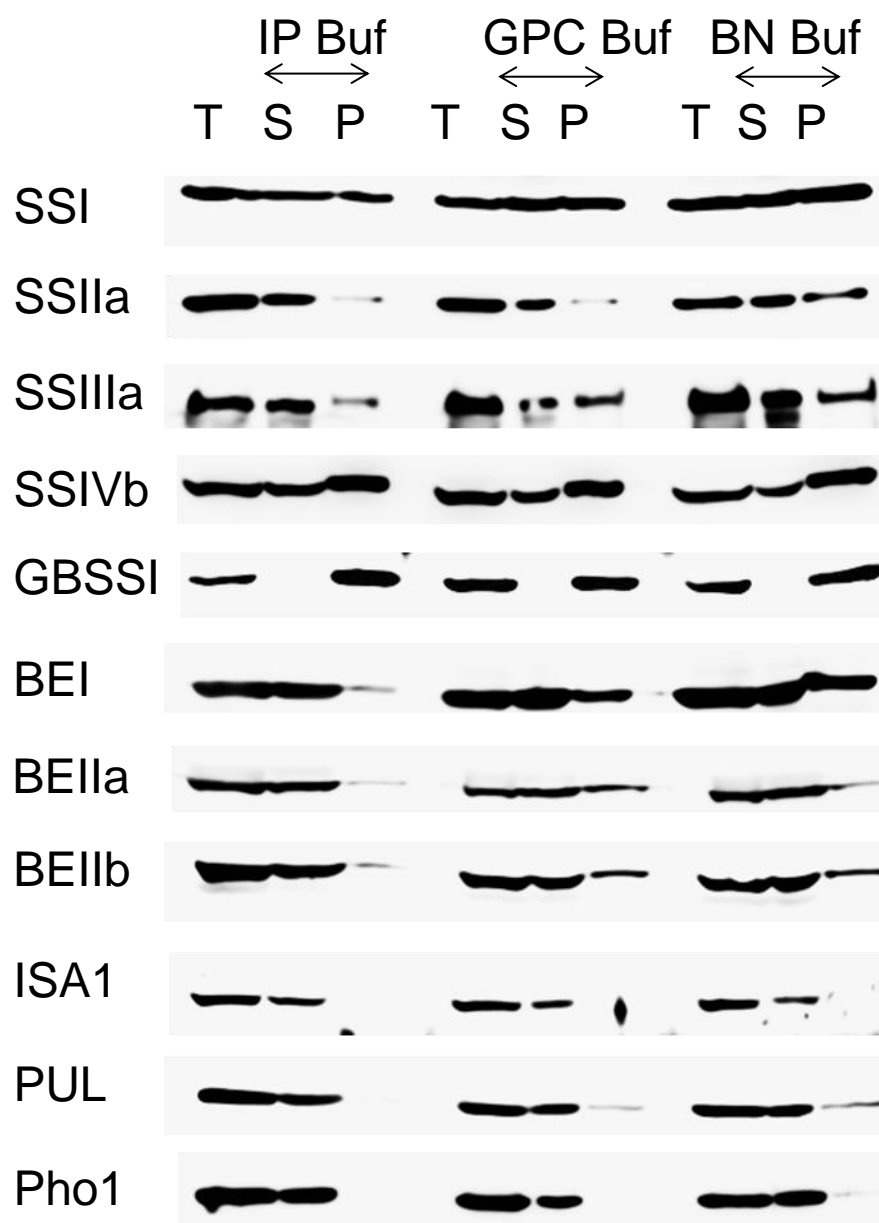**Fig. S2**

**A**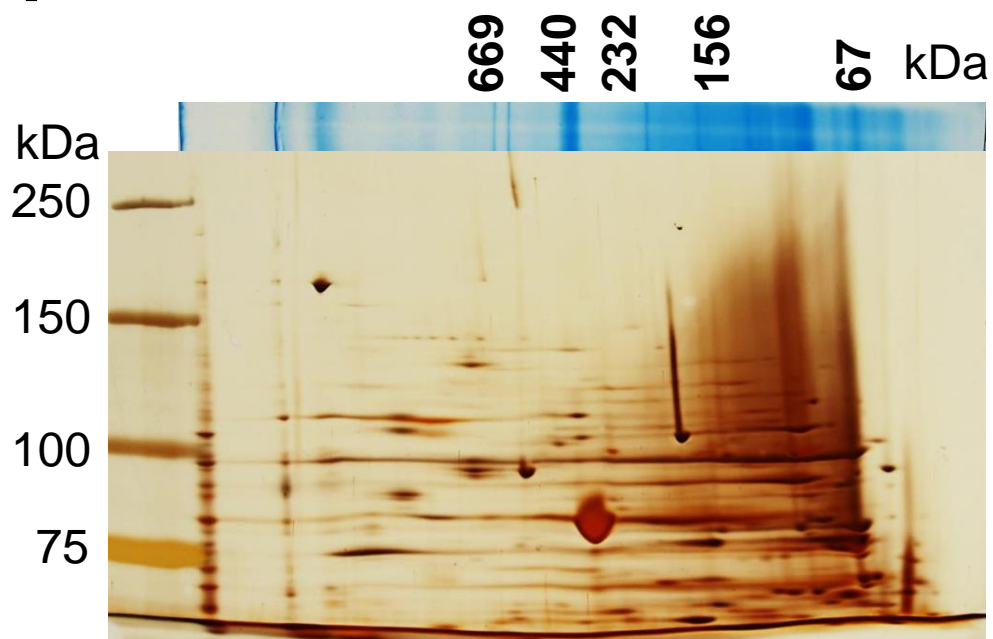**B**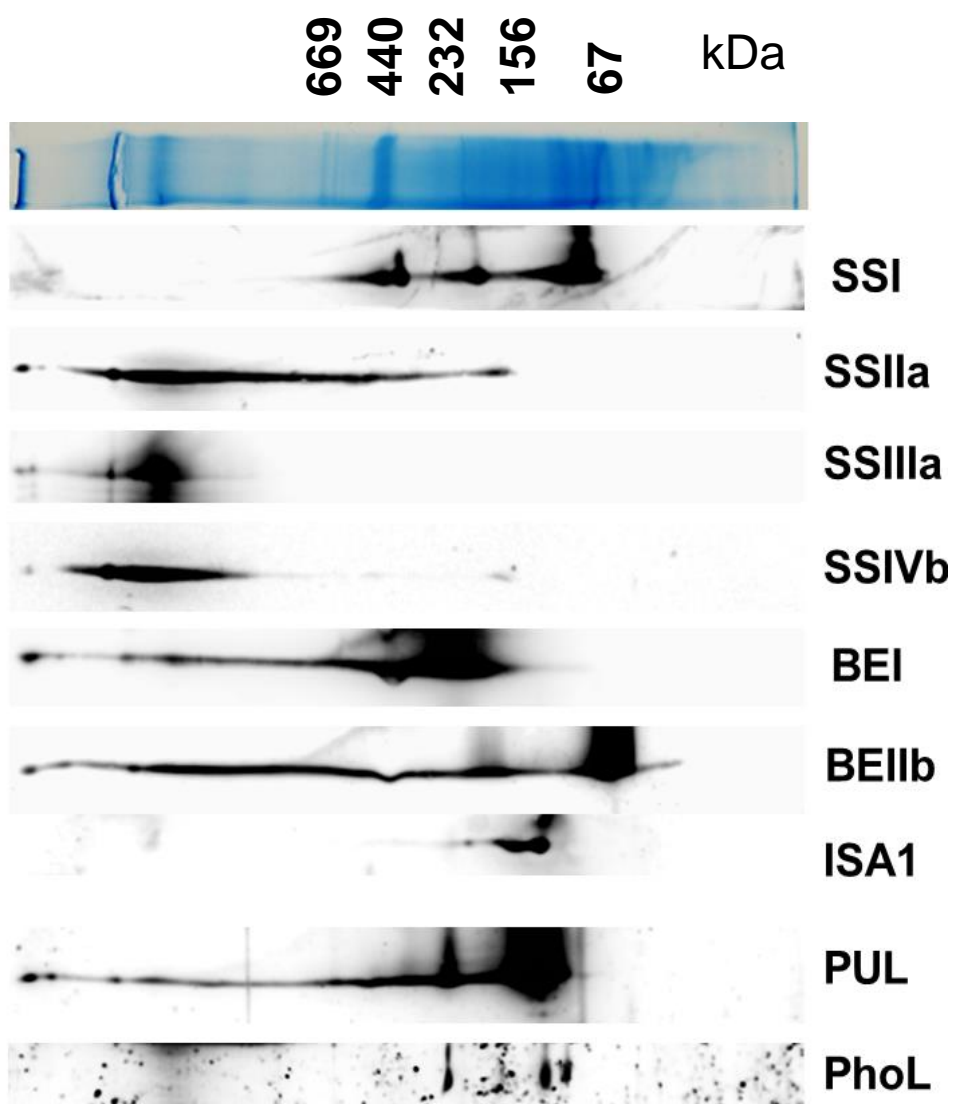**Fig. S3**

Supplement: Supplementary Data [file supp_erv212_jexbot146522_file001.pdf]
